# A Rare Case Report of a Vertebral Artery Variant Arising From the Aorta With Its Clinical Significance: An Uncommon Anatomy Unveiled

**DOI:** 10.7759/cureus.37716

**Published:** 2023-04-17

**Authors:** Mohamad Bakir, Ahmad Dawalibi, Asem H Elhossiny, Ayman Behiery

**Affiliations:** 1 College of Medicine, Alfaisal University, Riyadh, SAU; 2 Department of Anatomy, Alfaisal University, Riyadh, SAU

**Keywords:** vertebrobasilar system, vertebral artery dominance, extracranial vertebral artery, case report, variation, aortic arch origin, subclavian artery, vertebral artery, aberrant vessel, vertebral artery anomalies

## Abstract

The vertebrobasilar (VB) system, comprising two vertebral arteries and one basilar artery, is responsible for providing vital vascular supply to the central nervous system structures. Disruption in this network can lead to fatal neurologic outcomes, and variations in the origin of vessels may contribute to unexplained symptoms of clinical relevance. Therefore, an extensive understanding of the VB system's anatomy and its variations is crucial for diagnosing neurological disorders. Here, we report a case of a vertebral artery variant arising from the aortic arch proximal to the left subclavian artery in the cadaver of a 50-year-old male, discovered incidentally during a teaching dissection session. We also discuss the clinical pathophysiology and the relevance of the neurological symptoms in relation to the anomaly.

## Introduction

Two vertebral arteries (VA) and one basilar artery make up the vertebrobasilar system. During its course, the vertebral artery separates into four segments [1]. The first segment, also known as the V1 (preforaminal) segment, is formed above the first rib from the subclavian artery's first branch, and runs behind two muscles, the longus colli, and the anterior scalene [2]. The V2 (foraminal) segment, along with the descending vertebral venous plexus and sympathetic plexus, reaches the transverse foramen of the sixth cervical vertebrae [3]. The anterior spinal arteries of the VA supply the surrounding muscles at each cervical level. The V3 (Atlantic or extradural) segment travels laterally after departing the axis' transverse foramen and through C1's transverse foramen. The V4 (intracranial or intradural) segment passes across the suboccipital triangle into the intracranial area via the foramen magnum. Before combining with the contralateral VA to produce the basilar artery at the base of the pons, the VA gives out the posterior inferior cerebellar artery [4]. Embryologically, the VA begins to form between 33 and 55 days of development, from the longitudinal anastomoses of the first to seventh cervical intersegmental arteries, and progressing inferiorly to the sixth intersegmental artery [2,5]. As the seventh intersegmental artery forms into part of the subclavian artery and joins the primitive vertebral artery to create the posterior circulation system, the first to sixth intersegmental arteries disappear [2,6]. Whilst the left vertebral artery usually arises from the subclavian arteries, it can potentially originate from the aorta with a prevalence of 2.4-5.8% due to a faulty anastomosis between the sixth and seventh intersegmental arteries [2]. Such variations are associated with an increased risk of cerebrovascular events, vertebral artery dissection, and iatrogenic damage during carotid artery surgeries [6, 7]. VA variants are mostly benign, however, any alterations along the VA's course raise the chance of dissection or occlusion, and even increase the risk of Alzheimer's disease [4]. In this paper, we present a rare anomaly of a vertebral artery with a non-subclavian artery origin in a 50-year-old cadaver.

## Case presentation

**Figure 1:**
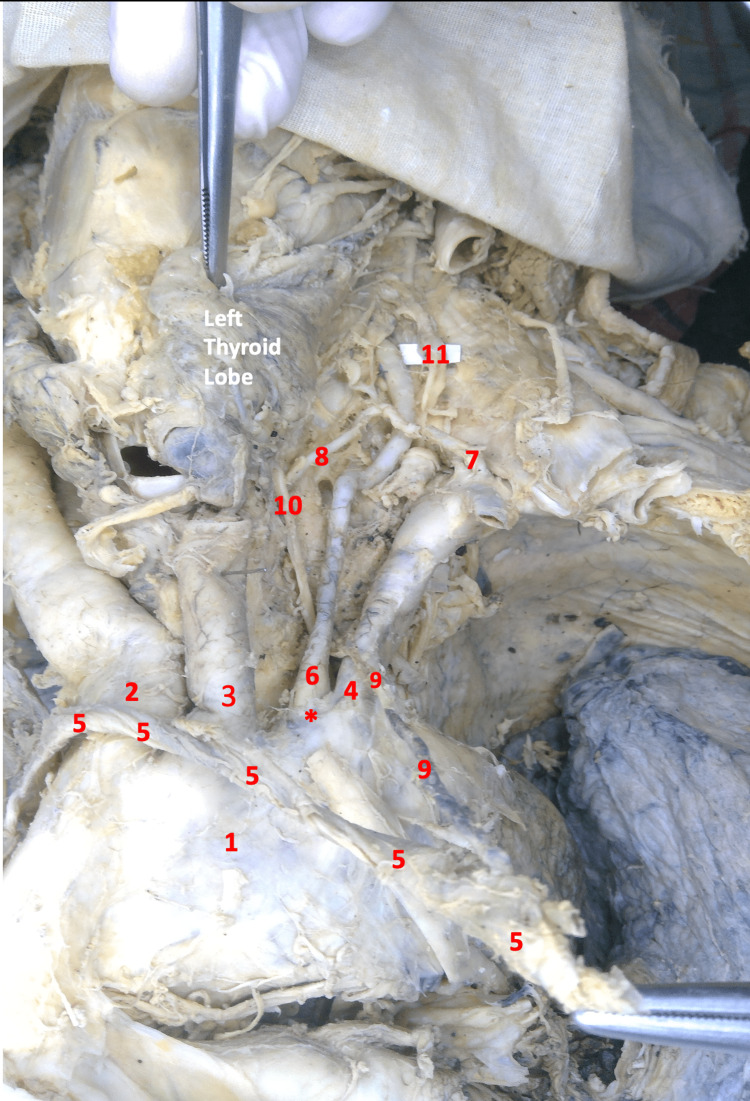
Image of the thoracic region and left neck in gross dissection 1- Aortic Arch, 2- Brachiocephalic Trunk, 3- Left Common Carotid Artery, 4- Left Subclavian Artery, 5- Left Brachiocephalic Vein (Mobilized), 6- Left Vertebral Artery, 7- Thyrocervical Artery, 8- Left Inferior Thyroid Artery, 9- Thoracic Duct (pulled by the mobilized venous angle), 10- Left Recurrent Laryngeal Nerve, 11- Left Sympathetic Trunk. *- Direct Origin of the Left Vertebral Artery from the Arch of Aorta (Anomalous Origin)

During a routine anatomy lab session, a mobilized thoracic part belonging to a formalin-fixed male cadaver of 50 years was dissected in the Department of Anatomy at Al-Faisal University's College of Medicine, Riyadh, Saudi Arabia. Throughout the whole process, Grant’s Dissector manual was used as a reference, and digital photos were taken of the entire area dissected. Initially, four branches from the arch of the aorta were noticed instead of the normal three: brachiocephalic trunk, left common carotid artery, and left subclavian artery. Following meticulous dissection, an anomalous origin of the left vertebral artery from the arch of the aorta was detected just proximal to (to the right of) the origin of the left subclavian artery. The anomalous origin of the left vertebral artery resulted in a deviation of the thoracic duct to run lateral to the artery itself instead of medial and anterior to it (Figure 1).

**Figure 2:**
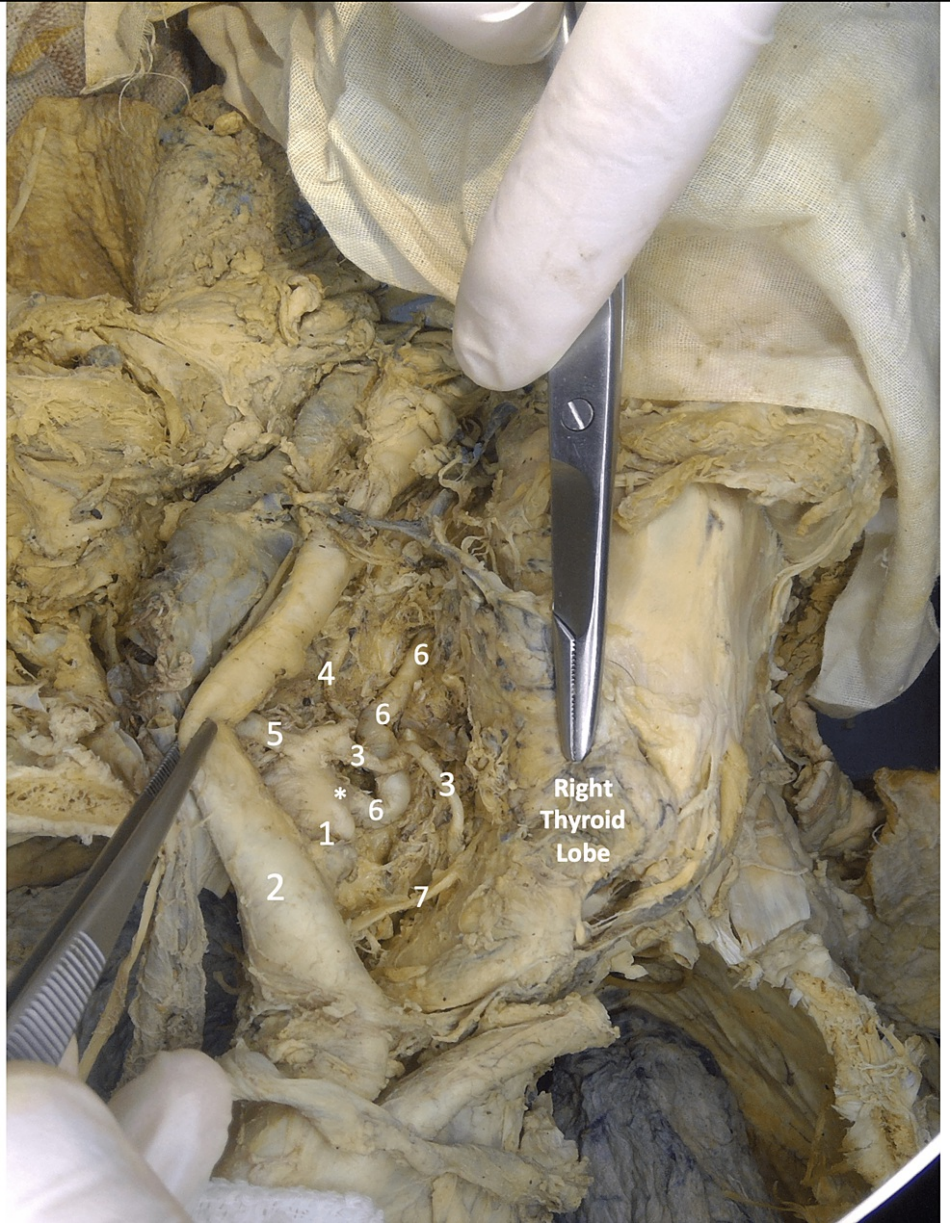
Image of the thoracic region and right neck in gross dissection 1- Right Subclavian Artery, 2- Right Common Carotid Artery, 3- Inferior Thyroid Artery, 4- Ascending Cervical Artery, 5- Common Stem for Transverse Cervical and Suprascapular Artery, 6- Right Vertebral Artery, 7- Right Recurrent Laryngeal Nerve *- Origin of Right Vertebral Artery from the Right Subclavian Artery

After confirming the abnormal origin of the left vertebral artery from the arch of the aorta, the neck dissection continued following the artery from its origin at the base of the Chassaignac's triangle to its apex: the Chassaignac’s triangle is bordered by the longus colli muscle medially, the scalenus anterior muscle laterally, with the subclavian artery forming its base. The apex of the triangle is formed by the tubercle of the sixth cervical vertebra (Chassaignac's tubercle). The left vertebral artery measured 9 cm in length with a diameter of 2.4 mm in the neck part (from the origin to the Chassaignac’s triangle). The rather lengthy part of the vessel, in addition to the small diameter arising from a high-pressure source (the arch of the aorta), makes the left vertebral artery prone to dissection and aneurysm. On finding this anomaly, we shifted the dissection to the right side of the neck to compare the right vertebral artery with the left (Figure 2).

**Figure 3:**
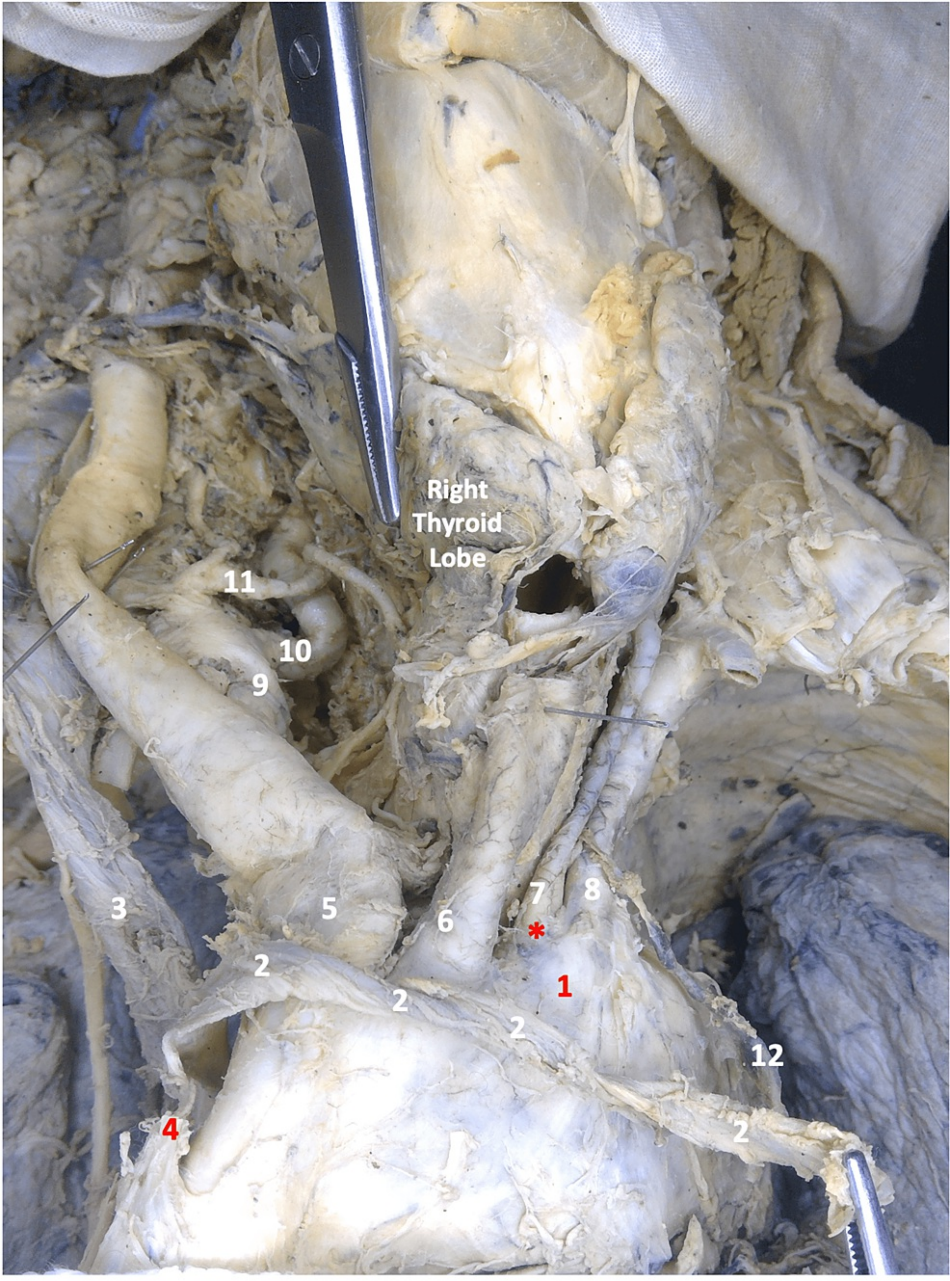
Image of the thoracic region and bilateral neck in gross dissection 1- Arch of Aorta, 2- Left Brachiocephalic Vein (Mobilized), 3- Right Brachiocephalic Vein, 4- Superior Vena Cava, 5- Brachiocephalic Trunk, 6- Left Common Carotid Artery, 7- Left Vertebral Artery, 8- Left Subclavian Artery, 9- Right Subclavian Artery, 10- Right Vertebral Artery, 11- Right Inferior Thyroid Artery, 12- Thoracic Duct (pulled by the mobilized venous angle) *- Direct Origin of the Left Vertebral Artery from the Arch of the Aorta (Anomalous Origin)

On the right side, the right vertebral artery appeared to be more tortuous and kinked with an accentuated curvature at its origin, and measuring 5.2 cm long, with a diameter of 4.45 mm. The size of the vertebral arteries can vary among individuals, and sometimes one artery can be larger than the other. This is called vertebral artery dominance. In our case, the doubled caliber size in the right vertebral artery compared to the left vertebral artery makes this case right-dominant instead of left-dominant, as the majority of the population tends to have the opposite (Figure 3).

This case illustrates an anatomical variation that should be considered during the management of cerebrovascular diseases.

## Discussion

Normally, the aortic arch gives rise to three vessels: the brachiocephalic trunk (BT), the left common carotid artery (LCC), and the left subclavian artery (LSA), however, variations to that have been identified and documented before [8]. In our paper, we discuss a rare variation, where a fourth branch, an aberrant left vertebral artery, emerges from the arch.

Vascular variations can result in changes in blood flow, which may increase the likelihood of developing an aneurysm in the VB system. Vascular abnormalities such as dural AV fistulas (DAVF), arteriovenous malformations (AVM), and occasionally, the presence of primitive carotid-vertebrobasilar connections are some examples of such irregularities [4]. Anatomical changes in the origin of the VA can possibly influence the cerebral hemodynamics in a variety of ways: 1) When originating from the aorta, the VA experiences greater stress from the direct pulsatile flow, while when originating from the subclavian artery, it experiences reduced blood flow through the proximal subclavian artery. 2) The level at which the VA enters the transverse foramen also plays a role; the VA originating from the aorta usually enters at a higher level (C5-6 intervertebral level) than the VA originating from the subclavian artery (C6-7 intervertebral level), which may result in more force in the distal portion of the VA, and predispose to injury during anterior cervical spinal surgery [4, 6].

Although vertebral artery injury is a rare consequence of spinal surgery, special precautions should be taken to prevent disrupting the vertebrobasilar system, which may cause massive bleeding as a consequence [9, 10]. For that reason, it is suggested to conduct computed tomography angiography before surgery if there is suspicion of a variant artery or in cases of traumatic cervical spine subluxations, due to the relatively high occurrence of VA variants [1]. Identifying and understanding abnormal origins of VA is most valuable for diagnostic purposes, before planning supra-aortic artery, aortic arch, and endovascular surgeries. On the other hand, it can also pose a risk in diagnosing lesions when it is missed or presumed to be obstructed during tests like MR angiography, CT angiography, and Doppler sonography, leading to diagnostic errors [11].

VB insufficiency is a transitory partial ischemia of the posterior circulation caused by reduced VB blood flow. One of the most common symptoms for VB insufficiency is vertigo, but individuals may also experience drop episodes or diplopia [12]. Atherosclerosis is the main cause of VB insufficiency [12]. A quick drop in blood pressure, such as postural alterations, or increased blood flow to the lower body, might set off an episode. Several studies have shown an increased risk of cerebrovascular accidents such as atherosclerosis, intracranial vessel malformations, arterial dissections, and the consequent intracranial complications owing to abnormal blood flow caused by the VAs' unusual origin. Atherosclerosis frequently affects the VA's extracranial section, constricting its root. This constriction, known as VA stenosis, is responsible for about 9% of transient ischemic attacks (TIA) and ischemic strokes in the posterior circulation [13].

A tear in the tunica intima of the vertebral artery causes a dissection. Dissections can be traumatic or spontaneous, with the latter being linked to connective tissue problems [14]. VA dissection symptoms include VB insufficiency, a dull or throbbing headache, dysphagia, dysarthria, visual loss, ataxia and a partial Horner's syndrome. When the VA arises from an abnormal origin, it runs through a longer extra-cranial path, exposing it to more shear stress, which may ultimately lead to an intimal tear. Therefore, in a patient with an abnormal VA origin, neurological complaints and headaches should prompt a complete evaluation to exclude dissection. A study by Komiyama et al. discovered a greater incidence of arterial dissection in the left VA originating from the aortic arch than both, the left VA from the left subclavian artery, and the right VA from the right subclavian artery [6]. While the cause of this increased prevalence with VA of aortic origin is not well understood, the longer course the VA has to go through to reach the C4 or C5 foramen transversarium may increase the likelihood of VA dissection [2, 6].

The subclavian steal phenomenon is caused by the VB system's redundancy. Atherosclerosis, a cervical rib, or Takayasu arteritis cause proximal stenosis of the subclavian artery, resulting in a high-resistance route for blood flow through the subclavian artery. Because of the low resistance distal to the occlusion, blood flows retrogradely down the ipsilateral VA to the afflicted upper extremity, resulting in a reduction of blood flow to the posterior circulation [15]. While subclavian steal syndrome is often asymptomatic, it may present with syncope or presyncope, neurologic impairments, a blood pressure disparity between arms, and unilateral arm claudication, especially during activities that involve the affected upper extremity [16].

Many detection methods can be used to evaluate the VB system for diagnostic purposes. Doppler ultrasonography was the first tool used to test the patency of the VAs' extracranial parts; however, it cannot visualize the origin of the VAs in some patients [2]. In the examination of the vertebral and basilar arteries, computed tomography, magnetic resonance angiography, and digital subtraction angiography (DSA) are currently the preferred diagnostic methods [17, 18].

The rationale for the case report is to highlight a variation in the vertebrobasilar (VB) system, specifically the origin of the vertebral artery from the aortic arch. The significance of understanding the anatomy and variations of the VB system cannot be overstated, as disruption of this network can have fatal neurologic outcomes. Accurate diagnosis and management of neurological disorders depend on a thorough knowledge of the VB system's anatomy and variations, and the ability to recognize and interpret abnormalities in imaging studies. Our case report aims to increase awareness of this anatomical variant and its potential clinical implications, and to contribute to the growing body of literature on variations in the VB system.

When evaluating our paper, we should keep our case report's limitations in mind. To begin, our report focuses on a single cadaver, therefore the findings may not be generalizable to the general population. Furthermore, case reports, including ours, are often regarded as low on the evidence hierarchy due to a lack of statistical power and control. As a result, our findings should be evaluated cautiously and in light of previous evidence. Despite these limitations, we feel our case report gives useful insights into a rare and interesting case, as well as emphasizes the need of knowing the variations in the vertebrobasilar system in relation to neurological conditions.

## Conclusions

The vertebral arteries are a pair of arteries that branch off the subclavian arteries and run up the cervical spine. Most people acquire their left vertebral artery from their left subclavian artery. However, in rare situations, the left vertebral artery may avoid the subclavian artery and arise directly from the aorta.

Although a non-subclavian origin of the left vertebral artery is rare, it is not always a cause for concern. Nonetheless, while performing certain procedures such as angiograms or surgeries in the neck and head region, medical professionals should be aware of this anatomical variance. The existence of this variation during angiography may influence picture interpretation, and catheterization of the vertebral artery may necessitate a modified method. To avoid damaging the left vertebral artery, surgeons may need to adjust their technique during some surgeries, such as carotid endarterectomy or neck dissection. To avoid challenges during diagnostic and therapeutic treatments, healthcare professionals including radiologists, surgeons, and interventionalists must be aware of the non-subclavian origin of the left vertebral artery.
